# The Savoy Baths

**Published:** 1909-05-22

**Authors:** 


### THE SAVOY BATHS.

The Savoy Turkish, Russian and Electric Baths, in Savoy Street, Strand, London, W.C., have recently, so we understand, undergone a change of proprietorship and a thorough redecoration and renewal of plant. We can confidently recommend these baths as they stand at the present time to the medical profession and the public. The various rooms are well equipped and decorated, and admirably clean, and the atmosphere is particularly fresh. Patients can safely be sent to them for the ordinary Turkish and Russian baths, which we have personally tried; or for the various forms of medicated and electric baths, the installation of which seemed to us to be all that it should be. The ventilation of the hot rooms of the Turkish bath is exceptionally good, and a visit of ordinary duration is not attended with the headache and lassitude often experienced. The shampooing and the general arrangements of the cooling rooms, etc., impressed us favourably, and the prices are moderate.

